# A Clinico-Epidemiological Study of Scrub Typhus Cases in a Tertiary Care Center of a Non-endemic Area

**DOI:** 10.7759/cureus.99339

**Published:** 2025-12-15

**Authors:** Mahendra Kumar Meena, Sourav Ranjan Parija, Shefali Gupta, Anirudh Mukherjee, Saubhagya Kumar Rout, Pramod Kumar

**Affiliations:** 1 General Medicine, All India Institute of Medical Sciences, Raebareli, Raebareli, IND; 2 Internal Medicine, All India Institute of Medical Sciences, Raebareli, Raebareli, IND; 3 Microbiology, All India Institute of Medical Sciences, Raebareli, Raebareli, IND

**Keywords:** acute undifferentiated febrile illness, india, mods, mortality predictors, non-endemic region, scrub typhus, seasonality

## Abstract

Background

Scrub typhus is a re-emerging mite-borne rickettsial infection, traditionally endemic to hilly regions with scrub vegetation, with an emerging spread into non-endemic areas. Given its common presentation as acute undifferentiated febrile illness (AUFI), it often remains underdiagnosed in these areas, even by astute physicians. The study was undertaken to evaluate the socio-epidemiological and clinical characteristics, including complications and mortality, of scrub typhus in a non-endemic region.

Methodology

This retrospective study included 64 patients diagnosed with scrub typhus using an IgM enzyme-linked immunosorbent assay. Medical records were reviewed for seasonal patterns, presentations, and predictors of mortality.

Results

The seroprevalence rate was 15.6% among the tested patients. Cases occurred predominantly between August and November. Common presentations included cough and dyspnea (43.8%), gastrointestinal symptoms (26.5%), and altered sensorium (14.0%). Eschar was observed in only 4.6% of cases. Multiple organ dysfunction syndrome developed in 37.5%, with one-third mortality among these patients. Acute respiratory distress syndrome occurred in 26.5%. Laboratory abnormalities included transaminitis (59.3%), jaundice (28.0%), and acute kidney injury (26.5%). Overall mortality was 14.0%. Encephalopathy and shock were independent predictors of mortality.

Conclusions

Scrub typhus in non-endemic regions presents with significant multi-organ involvement. Low eschar frequency and diverse presentations contribute to diagnostic challenges. Early empirical doxycycline or azithromycin therapy should be considered for AUFI patients to prevent complications from delayed diagnosis.

## Introduction

Most patients with acute undifferentiated febrile illness (AUFI) by definition present with overlapping symptoms such as fever, chills, headache, and myalgia without localizing signs, making accurate diagnosis challenging given the wide spectrum of potential etiologies. Thus, knowledge of endemicity and seasonal trends is crucial for clinicians to narrow down the differential diagnoses [1]. Scrub typhus is one such disease, caused by *Orientia tsutsugamushi*, an obligate intracellular, rickettsial Gram-negative coccobacillus transmitted to humans through the bite of chiggers, the larval stage of trombiculid mites [2-4]. Classically, it is endemic to the tsutsugamushi triangle, encompassing large parts of South and Southeast Asia and extending to northern Australia, including India. However, recent reports from South America, Africa, and the Middle East suggest a wider distribution, highlighting its emergence as a global health concern [5,6]. In India, scrub typhus was first documented during World War II among field troops. The reported incidence subsequently declined over the following decades, possibly due to widespread use of tetracyclines and chloramphenicol, with persistence of endemicity largely restricted to South India and the sub-Himalayan belt [7,8]. However, in recent years, multiple epidemics and sudden outbreaks have been documented by the National Centre for Disease Control across various regions of India, establishing scrub typhus as a re-emerging infection [9,10].

*O. tsutsugamushi* primarily targets endothelial cells and macrophages through its unique 56-kDa type-specific antigen (TSA). This mechanism leads to endothelial dysfunction and acute vasculitis [11]. The clinical spectrum ranges from self-limiting febrile illness to severe multi-organ dysfunction with potentially fatal outcomes. Disease severity increases substantially when cases remain undiagnosed or untreated. In low- and middle-income countries such as India, diagnostic capacity faces significant constraints. Overburdened healthcare systems and unequal access to laboratory testing compound these challenges. Variable antibiotic resistance among different *O. tsutsugamushi* strains further complicates management. These factors collectively result in suboptimal or delayed treatment, thereby escalating complications, morbidity, and mortality. Without timely intervention, mortality rates demonstrate considerable variation across studies. The median mortality approximates 6%, with a broad range from 0% to 70% [12]. Even with appropriate antibiotic therapy, in-hospital mortality reaches up to 9% in South Indian studies [11].

This study evaluated seasonality, demographic characteristics, clinical profiles, outcomes, and mortality predictors in emerging scrub typhus cases at a tertiary care hospital in Uttar Pradesh. The findings aim to enhance clinical decision-making in non-endemic regions.

## Materials and methods

This hospital-based, retrospective, cross-sectional study was conducted at the All India Institute of Medical Sciences, Raebareli, Uttar Pradesh, between September 2023 and November 2024. Patients aged >16 years presenting with fever were recruited from the medicine outpatient department, emergency services, inpatient wards, or intensive care unit. Only those testing positive for IgM scrub typhus antibody by enzyme-linked immunosorbent assay (ELISA) were included. Patients with IgG antibody positivity or travel history to scrub typhus endemic regions were excluded. The study was undertaken to evaluate the socio-epidemiological and clinical characteristics, including complications and mortality, of scrub typhus at a non-endemic region. Data from 64 eligible cases were collected, tabulated, and analyzed using SPSS version 27 (IBM Corp., Armonk, NY, USA). The study was approved by the Institutional Ethics Committee, AIIMS, Raebareli (approval number: 2024-15-OTH-EXP-9).

Serological confirmation utilized the detection of IgM antibodies against the 56-kDa TSA of *O. tsutsugamushi*. A commercial IgM ELISA kit (Biogenix) was employed according to the manufacturer’s instructions. Clinical data encompassed history, physical examination findings, and laboratory parameters extracted from medical records. Laboratory investigations comprised complete blood count, liver and renal function tests, serum electrolytes, erythrocyte sedimentation rate, C-reactive protein, and procalcitonin based on availability. Urine analysis was performed routinely. Given overlapping clinical features with other AUFIs, additional diagnostic tests for malaria, dengue, leptospirosis, and typhoid were conducted when clinically indicated.

Systemic involvement in scrub typhus was defined using standardized criteria. Pneumonia was diagnosed based on radiological evidence of alveolar or interstitial infiltrates without features of pulmonary edema. Acute respiratory distress syndrome (ARDS) required acute-onset non-cardiogenic pulmonary edema with bilateral infiltrates on chest radiograph and PaO₂/FiO₂ ratio <300. Acute kidney injury (AKI) was defined as a serum creatinine rise of ≥0.3 mg/dL or ≥50% above baseline within 48 hours, or urine output of <0.5 mL/kg/hour for at least six hours. Transaminitis was established when levels exceeded twice the upper limit of normal. Hyperbilirubinemia was defined as total serum bilirubin >2 mg/dL. Acute febrile encephalopathy encompassed confusion and decreased Glasgow Coma Scale score secondary to infection or metabolic derangement [13]. Multiple organ dysfunction syndrome (MODS) requires potentially life-threatening dysfunction involving two or more organ systems, defined by a two-point gain in any lineage of the Sequential Organ Failure Assessment score [14].

Normality of continuous variables was assessed using the Shapiro-Wilk and Kolmogorov-Smirnov tests. Categorical variables were analyzed using the chi-square test. Continuous variables were compared using the independent t-test for parametric data and the Mann-Whitney U test for non-parametric data. Odds ratios were calculated for potential predictors of complications. Statistically significant variables from univariate analysis were entered into multivariate logistic regression models. This approach identified independent predictors of unfavorable outcomes. Statistical significance was defined as a two-tailed p-value ≤0.05.

## Results

**Figure 1:**
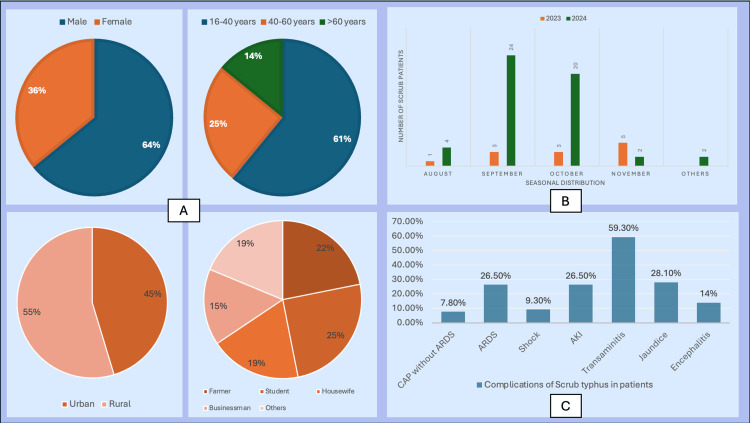
Socio-demographic parameters and complications in scrub typhus patients (n = 64). (A) Age, sex, residency, and occupation of patients. (B) Seasonal distribution of patients. (C) Different complications at presentation or during hospital stay.

A total of 64 patients were diagnosed with scrub typhus during the study period, which included 16 cases in 2023 and 48 in 2024. Peak incidence occurred in September (42.1%) and October (34.9%). The mean age was 39.4 years, with 61% younger than 40 years. Males comprised 64% of the cohort. Eschars were identified in only three (4.7%) patients. Data regarding rash and lymphadenopathy were unavailable from medical records. Most cases (71.8%) were residents of Raebareli, with the remainder from adjoining districts. Urban areas accounted for 45.3% of patients. 25% of all patients were students, while nearly 22% were farmers, and 18.7% were housewives, as shown in Figure 1.

All patients presented with fever. The mean duration at presentation was 8.77 ± 3.96 days. The most common associated symptoms were chills and rigor (59.4%), followed by breathlessness and headache (35.9% each), abdominal pain and diarrhea (26.6%). Jaundice affected 28.1%, while altered sensorium occurred in 14%. The most frequent complications were transaminitis (59.3%). ARDS and AKI each affected 26.5% of patients. No diagnosed cardiac complications, such as myocarditis and atrial fibrillation, were documented. MODS occurred in 37.5% of patients, of whom one-third died. Overall, 22 patients required intensive care, including 18 who needed mechanical ventilation. The overall mortality rate was 14%. Septic shock developed in six (9.3%) patients. All of them succumbed despite prompt management. Doxycycline served as the treatment mainstay, either as monotherapy or combined with azithromycin or rifampicin, demonstrating favorable clinical outcomes in survivors.

As shown in Table 1, leukocytosis was observed in 42% of patients. Leukopenia occurred in 6.25%, while the remainder exhibited normal leukocyte counts. Thrombocytopenia was present in 76.5% of cases. Hyponatremia and hyperuricemia were documented in 51.5% and 46.6% of patients, respectively. Albuminuria was noted in 28 (43.7%) patients, though four of them had other identifiable causes of proteinuria. However, it did not show any significant association with complications. Co-infections were detected in a subset of patients. Three tested positive for malaria, three for dengue, and four for *Leptospira* IgM. An additional seven had equivocal *Leptospira* seropositivity. Co-infections did not significantly impact mortality.

**Table 1:** Deranged laboratory parameters in scrub typhus patients. CRP = C-reactive protein; ESR = erythrocyte sedimentation rate

Laboratory parameters	Total, n (%)
Moderate to severe anemia (<10 g/dL)	16 (25%)
Leukopenia (<4,000/μL)	4 (6.25%)
Leucocytosis (>11,000/μL)	27 (42.2%)
Thrombocytopenia (<150,000/μL)	49 (76.5%)
Severe thrombocytopenia (<50,000/μL)	16 (25%)
Hyponatremia (<135 mmol/L)	33 (51.5%)
Elevated creatinine (>2 mg/dL)	9 (14%)
Hyperuricemia (>7 mg/dL)	26 (40.6%)
Transaminitis (>100 IU/L)	31 (48.4%)
Hyperbilirubinemia (>2 mg/dL)	21 (32.8%)
Hypoalbuminemia (<3.5 mg/dL)	48 (75%)
Albuminuria	28 (43.7%)
Elevated ESR (>30 mmHg) (n = 33)	16 (48.5%)
Elevated CRP (>0.5 mg/dL) (n = 32)	32 (100%)
Elevated procalcitonin (>0.5 ng/dL) (n = 39)	32 (82.1%)

**Figure 2:**
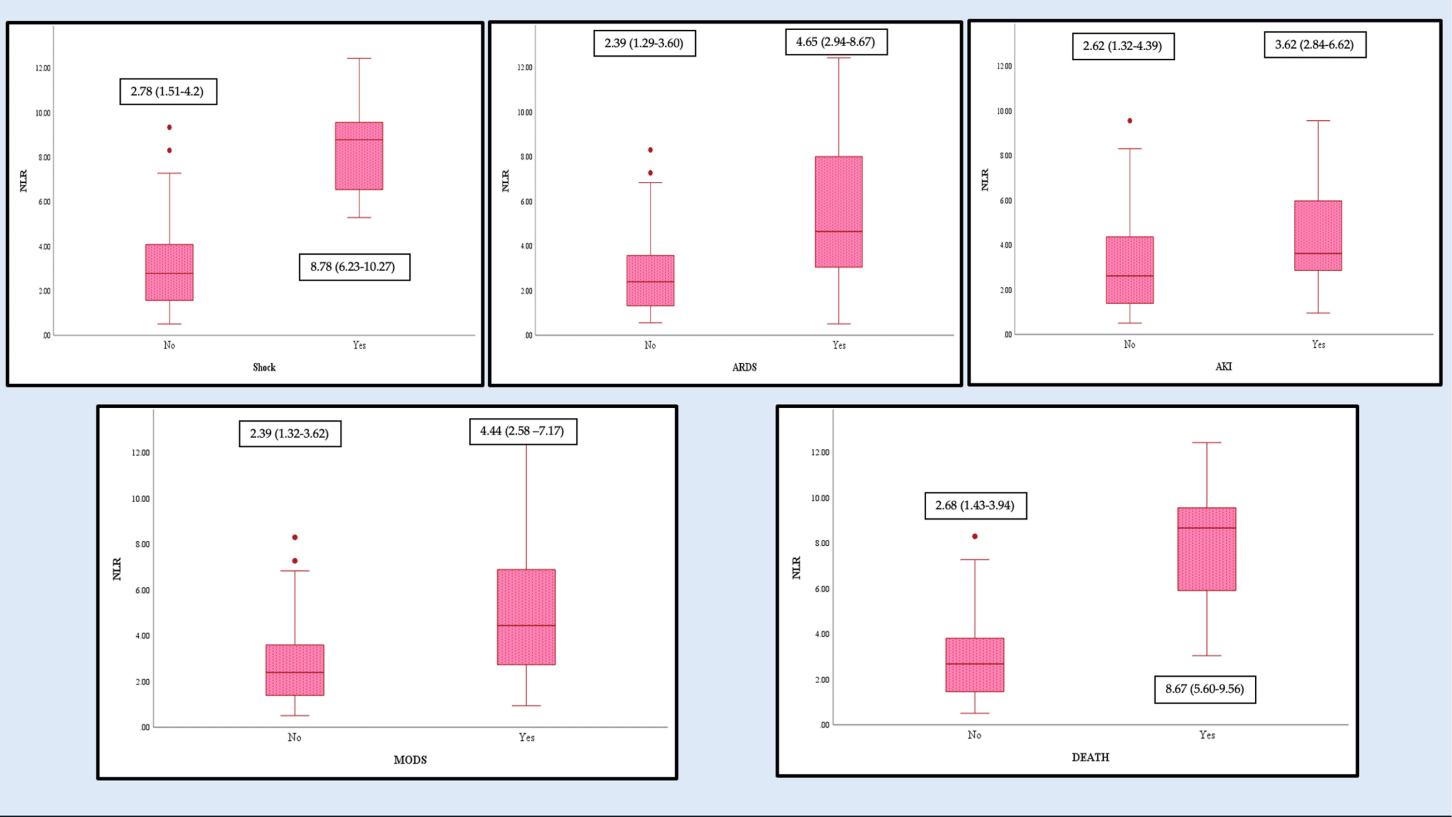
Significant association of NLR in complications such as shock (p < 0.005), ARDS (p = 0.004), AKI (p = 0.011), MODS (p = 0.008) and mortality (p < 0.005) of scrub typhus patients. Median (IQR) is mentioned with each box and whisker plot. Results are expressed as median (IQR). P-value was calculated using the Mann-Whitney U test. ARDS = acute respiratory distress syndrome; AKI = acute kidney injury; MODS: multi-organ dysfunction syndrome; NLR = neutrophil-to-lymphocyte ratio

As shown in Table 2, univariate analysis revealed age as a significant risk factor for ARDS (p = 0.023) and AKI (p = 0.021). Multivariate analysis demonstrated that patients aged 40-60 years had a 5.5-fold higher risk of developing AKI compared to those <40 years. Male patients were less likely to develop shock (OR = 0.09, 95% confidence interval (CI) = 0.01-0.827) and ARDS (OR = 0.268, 95% CI = 0.084-0.852), although these associations did not remain significant in multivariate analysis. Thrombocytopenia was associated with jaundice (p = 0.003), transaminitis (p = 0.02), and AKI (p = 0.011). Leukocytosis correlated with shock (p = 0.003), ARDS (p = 0.008), and AKI (p = 0.031). Neutrophil-to-lymphocyte ratio (NLR) was significantly elevated in all complications except hepatic involvement and encephalopathy (Figure 2, Tables 3-5).

**Table 2:** Predictor of complications and mortality after logistic regression in patients with scrub typhus. Results are expressed as OR (95% CI). *: statistically significant associations. Odds ratio and p-value have been calculated using the logistic regression analysis using models involving all the parameters included under each complications separately. ARDS = acute respiratory distress syndrome; AKI = acute kidney injury; MODS: multi-organ dysfunction syndrome; NLR = neutrophil-to-lymphocyte ratio; OR = odds ratio; CI = confidence interval

Predictors	Univariate OR	95% CI (lower–upper)	Adjusted OR	95% CI (lower—upper)
Death				
Shock (n = 6)	206.14*	18.61–29,090.1	74.02*	2.28–30,639.6
Encephalitis (n = 9)	36.67*	5.67–211.77	64.38*	4.57–15,231.9
ARDS (n = 17)	40.89*	4.53–368.35	8.39	0.27–1,908.7
MODS (n = 24)	19.5*	2.25–168.88	1.06	0.008–79.4
AKI (n = 17)	4.48*	1.04–19.33	7.55	0.37–1,592.2
AKI				
Age (40–60)	5.5*	1.48–20.39	8.777*	1.511–50.985
Uric acid (>7)	13.61*	3.32–55.68	19.839*	3.610–109.036
MODS				
NLR 3–7	2.85	0.91–9.41	6.87*	1.53–43.40
NLR 7–17	10.2*	2.18–64.8	89.63*	4.44–22,547.3
Albumin <3.5 mg/dL	5.91*	1.21–28.57	4.76	0.92–33.33
ARDS				
Sex (male)	0.27*	0.08–0.85	0.36	0.11–1.19
NLR 7–17	11.76*	2.45–68.68	9.71*	1.95–57.94
Shock				
Male	0.09*	0.01–0.83	0.17	0.01–1.11
NLR 7–17	53.18*	4.7–7,438.82	41.12*	3.35–5,890.1
Encephalitis				
Uric acid	6.63*	1.25–35.12	4.83*	1.08–29.11

**Table 3:** Basic patient characteristics and association with mortality (n = 64). Results are expressed as mean ± SD and n (%) as and when necessary. *: statistically significant associations. ^a^: P-value has been calculated using the independent t-test and the Mann-Whitney U test as per the normal and non-normal distribution of data, respectively. ^b^: P-value has been calculated using the chi-square analysis. ^: t value is given. Note: The entities without ^ marks in the column named (t-value/u-value) are u-values with a non-normal distribution. NLR = neutrophil-to-lymphocyte ratio; CRP = C-reactive protein; ESR = erythrocyte sedimentation rate; PCT = procalcitonin; AST = aspartate transaminase; ALT = alanine transaminase; ALP = alkaline phosphatase; GGT = gamma glutamyl transferase; LDH = lactate dehydrogenase; BUN/Cr = blood urea nitrogen to creatinine ratio

Patients’ parameters	Total	Alive	Dead	t-value/u-value	P-value
Age in years (mean ± SD)	39.42 ± 17.10	39.75 ± 16.56	37.44 ± 21.12	0.372^	0.711^a^
Sex					
Male, n (%)	41 (64.1)	38 (92.7)	3 (7.3)	-	0.038*^,b^
Female, n (%)	23 (35.9)	17 (73.9)	6 (26.1)		
Signs and symptoms					
Duration of fever presentation (mean ± SD)	8.77 ± 3.96	8.75 ± 3.80 (8.00)	8.89 ± 5.11 (7.00)	227.00	0.688^b^
Day of stay in the hospital (mean ± SD)	7.14 ± 6.14	7.49 ± 6.32 (7.00)	5.00 ± 4.64 (3.00)	200.00	0.356^b^
Fever, n (%)	64 (100.0)	55 (85.9)	9 (14.1)	-	-
Chills, n (%)	38 (59.4)	31 (81.6)	7 (18.4)	-	0.225^b^
Headache, n (%)	23 (35.9)	21 (91.3)	2 (8.7)	-	0.355^b^
Dyspnoea, n (%)	23 (35.9)	15 (65.2)	8 (34.8)	-	0.000*^,b^
Altered sensorium, n (%)	9 (14.1)	3 (33.3)	6 (66.7)	-	0.000*^,b^
GI symptoms, n (%)	17 (26.6)	16 (94.1)	1 (5.9)	-	0.258^b^
Eschar, n (%)	3 (4.7)	3 (100.0)	0 (0.0)	-	0.473^b^
Biochemical parameters, n (%)					
Hemoglobin (mean ± SD)	11.09 ± 2.26	11.09 ± 2.27	11.08±2.34	0.016	0.988^a^
Total leukocyte count (mean ± SD)	10.95 ± 5.61	9.91 ± 4.81	17.27±6.30	-4.07^	0.008*^,a^
Neutrophil (mean ± SD)	67.48 ± 14.59	65.07 ± 14.07	82.22 ± 7.45	-5.49^	0.001*^,a^
Lymphocyte (mean ± SD)	25.81 ± 13.72	28.29 ± 13.07 (25.00)	10.67 ± 5.32 (9.00)	7.05^	0.000*^,a^
NLR (mean ± SD)	4.17 ± 4.35	3.14 ± 2.25 (2.75)	10.45 ± 7.94 (9.33)	42.00	0.000*^,a^
Platelets (mean ± SD)	120.81 ± 109.02	126.69 ± 114.70 (101.00)	84.89 ± 55.70 (60.00)	198.00	0.339^a^
CRP (n = 32) (mean ± SD)	14.53 ± 7.75	14.57 ± 8.17 (14.92)	14.39 ± 6.17 (16.54)	71.00	0.760^a^
ESR (n = 33) (mean ± SD)	43.36 ± 34.50	47.03 ± 34.82 (37.00)	16.75 ± 17.48 (10.00)	23.00	0.055^a^
PCT (n = 39) (mean ± SD)	6.70 ± 13.11	6.39 ± 14.28 (1.38)	8.12 ± 5.61 (8.25)	58.00	0.049*^,a^
Total bilirubin (mean ± SD)	2.02 ± 2.03	1.92 ± 1.93 (1.05)	2.66 ± 2.63 (1.69)	212.00	0.493^a^
AST (mean ± SD)	171.70 ± 210.33	139.99 ± 142.03 (95.00)	365.51 ± 403.78 (174.00)	159.00	0.087^a^
ALT (mean ± SD)	105.10 ± 117.91	86.40 ± 63.83 (71.00)	219.37 ± 254.72 (81.00)	218.00	0.569^a^
ALP (mean ± SD)	237.47 ± 192.57	240.89 ± 202.28 (162.00)	216.56 ± 123.61 (160.00)	236.00	0.824^a^
GGT (mean ± SD)	129.17 ± 89.09	133.62 ± 92.39 (117.00)	101.94 ± 62.58 (79.00)	202.50	0.385^a^
Serum albumin (mean ± SD)	2.93 ± 0.68	2.99 ± 0.69	2.57 ± 0.54	1.731	0.061^a^
LDH (mean ± SD)	1,138.19 ± 719.91	1,037.07 ± 455.17 (1,009.00)	1,756.11 ± 1,477.52 (1,200.00)	151.00	0.063^a^
Urea (mean ± SD)	54.52 ± 41.64	46.69 ± 28.78 (42.90)	102.40 ± 71.18 (77.10)	101.00	0.005*^,a^
Creatinine (mean ± SD)	1.40 ± 1.16	1.19 ± 0.57 (1.04)	2.71 ± 2.49 (1.78)	168.00	0.125^a^
BUN/Cr (mean ± SD)	20.51 ± 9.81	19.78 ± 8.84 (18.72)	24.98 ± 14.29 (20.92)	201.00	0.369^a^
Uric acid (mean ± SD)	6.60 ± 2.71	6.36 ± 2.60 (5.60)	8.04 ± 3.08 (9.30)	143.00	0.044*^,a^
Sodium (mean ± SD)	134.02 ± 4.55	133.96 ± 3.78	134.36 ± 8.14	-0.14^	0.812^a^
Potassium (mean ± SD)	4.34 ± 0.76	4.26 ± 0.63	4.79 ± 1.25	-1.24^	0.053^a^
Calcium (mean ± SD)	8.50 ± 0.83	8.57 ± 0.70 (8.40)	8.03 ± 1.37 (8.20)	177.00	0.173^a^

**Table 4:** Association of major laboratory parameters with complications in scrub typhus (part 1). Results are expressed as mean ± SD (median). *: statistically significant associations. P-value has been calculated using the independent t-test and the Mann-Whitney U test as per the normal and non-normal distribution of data, respectively. ^: t-value is given. Note: The entities without ^ marks in the column named (t-value/u-value) are u-values with a non-normal distribution. NLR = neutrophil-to-lymphocyte ratio; CRP: C-reactive protein; ESR: erythrocyte sedimentation rate; LDH: lactate dehydrogenase; TLC = total leukocyte count

	Shock				Jaundice				Encephalopathy			
	Yes	No	t-value/u-value	P-value	Yes	No	t-value/u-value	P-value	Yes	No	t-value/u-value	P-value
	Mean ± SD (median)	Mean ± SD (median)			Mean ± SD (median)	Mean ± SD (median)			Mean ± SD (median)	Mean ± SD (median)		
Age	33.67 ± 18.77 (32.50)	40.02 ± 16.98 (36.50)	131.50	0.336	36.06 ± 13.50 (36.50)	40.74 ± 18.28 (36.50)	0.99^	0.328	43.11 ± 22.41 (40.00)	38.82 ± 16.25 (36.00)	-0.70^	0.489
Platelet	74.33 ± 54.97 (54.50)	125.62 ± 112.34 (101.50)	122.00	0.242	67.61 ± 40.90 (50.50)	141.63 ± 120.13 (102.00)	215.00	0.003*	77.56 ± 55.31 (58.00)	127.89 ± 114.24 (102.00)	180.00	0.192
TLC	17.22 ± 7.64 (16.46)	10.30 ± 5.02 (9.35)	-3.06^	0.003*	12.63 ± 7.08 (12.74)	10.29 ± 4.86 (9.27)	-1.52^	0.134	11.10 ± 7.00 (10.10)	10.92 ± 5.43 (9.57)	-0.09^	0.929
NLR	8.56 ± 2.53 (8.78)	3.72 ± 4.26 (2.82)	27.00	0.000*	3.82 ± 2.70 (3.08)	4.31 ± 4.87 (2.98)	408.50	0.935	8.58 ± 9.05 (6.54)	3.45 ± 2.48 (2.84)	149.50	0.058
ESR	16.75 ± 17.48 (10.00)	47.03 ± 34.82 (37.00)	23.00	0.055	32.78 ± 34.74 (15.00)	47.33 ± 34.29 (38.50)	71.00	0.142	23.50 ± 26.16 (23.50)	44.65 ± 34.91 (30.00)	19.00	0.417
LDH	1,723.00 ± 1,739.03 (1,110.50)	1,077.69 ± 517.52 (1,023.00)	141.50	0.464	1,381.06 ± 1,070.55 (1,118.50)	1,043.15 ± 509.70 (946.00)	294.00	0.072	1,319.56 ± 790.42 (1,200.00)	1,108.51 ± 711.14 (1,017.00)	182.50	0.208
CRP	14.06 ± 6.84 (17.08)	14.62 ± 8.02 (15.69)	61.00	0.763	15.72 ± 8.81 (17.08)	13.91 ± 7.28 (12.90)	-0.62^	0.539	16.33 ± 3.91 (16.00)	14.35 ± 8.06 (15.69)	34.00	0.58
Procalcitonin	7.76 ± 6.05 (7.71)	6.51 ± 14.07 (1.38)	59.00	0.126	11.39 ± 23.02 (3.66)	5.30 ± 8.37 (1.46)	101.50	0.269	10.58 ± 9.74 (8.50)	5.85 ± 13.72 (1.38)	63.00	0.076
Uric acid	7.10 ± 3.41 (7.30)	6.55 ± 2.66 (6.05)	142.50	0.478	6.13 ± 2.72 (5.50)	6.78 ± 2.72 (6.10)	356.00	0.386	7.93 ± 2.95 (9.10)	6.38 ± 2.64 (5.60)	151.00	0.062
Albumin	2.37 ± 0.28 (2.35)	2.99 ± 0.69 (2.90)	4.27^	0.000*	2.43 ± 0.33 (2.38)	3.13 ± 0.68 (3.05)	5.54^	0.000*	2.87 ± 0.64 (2.53)	2.94 ± 0.69 (2.87)	0.29^	0.777

**Table 5:** Association of major laboratory parameters with complications in scrub typhus (part 2). NLR: Neutrophil to Lymphocyte Ratio, CRP: C-Reactive Protein, ESR: Erythrocyte Sedimentation Rate, LDH: Lactate Dehydrogenase, TLC: Total Leukocyte Count Results are expressed as mean ± SD (median). *: statistically significant associations. P-value has been calculated using the independent t-test and the Mann-Whitney U test as per the normal and non-normal distribution of data, respectively. ^: t value is given. Note: The entities without ^ marks in the column named (t-value/u-value) are u-values with a non-normal distribution. NLR = neutrophil-to-lymphocyte ratio; CRP: C-reactive protein; ESR: erythrocyte sedimentation rate; LDH: lactate dehydrogenase; TLC = total leukocyte count

	ARDS				AKI				MODS			
	Yes	No	t-value/u-value	P-value	Yes	No	t-value/u-value	P-value	Yes	No	t-value/u-value	P-value
	Mean ± SD (median)	Mean ± SD (median)			Mean ± SD (median)	Mean ± SD (median)			Mean ± SD (median)	Mean ± SD (median)		
Age	31.41 ± 12.97 (29.00)	42.32 ± 17.60 (38.00)	2.33^	0.023*	47.53 ± 15.95 (51.00)	36.49 ± 16.70 (34.00)	-2.36^	0.021*	36.29 ± 14.61 (35.50)	41.30 ± 18.35 (37.00)	1.14^	0.260
Platelet	99.00 ± 88.56 (54.00)	128.70 ± 115.37 (102.00)	307.50	0.162	73.88 ± 56.59 (60.00)	137.79 ± 118.55 (103.00)	233.00	0.011*	60.00 ± 38.84 (50.50)	157.30 ± 121.11 (126.50)	167.00	0.000*
TLC	14.87 ± 7.00 (14.07)	9.53 ± 4.29 (9.00)	-2.95^	0.008*	13.45 ± 6.69 (14.22)	10.04 ± 4.95 (9.38)	-2.21^	0.031*	12.47 ± 6.76 (12.15)	10.04 ± 4.66 (9.35)	-1.56^	0.128
NLR	5.65 ± 3.26 (4.65)	3.64 ± 4.60 (2.62)	209.00	0.004*	6.60 ± 6.89 (4.44)	3.29 ± 2.54 (2.62)	233.00	0.011*	5.28 ± 3.49 (4.44)	3.50 ± 4.71 (2.50)	290.00	0.008*
ESR	37.00 ± 33.40 (20.00)	45.75 ± 35.30 (33.50)	91.00	0.512	30.88 ± 34.73 (12.50)	47.36 ± 34.15 (40.00)	60.50	0.098	35.15 ± 35.68 (15.00)	48.70 ± 33.53 (38.50)	86.50	0.110
LDH	1,428.94 ± 1,137.89 (1,121.00)	1,033.02 ± 465.73 (1017.00)	316.50	0.206	1,535.71 ± 1,104.86 (1,121.00)	994.40 ± 453.54 (924.00)	238.50	0.014*	1,366.29 ± 1,003.57 (1,114.50)	1,001.33 ± 438.20 (907.00)	347.50	0.065
CRP	13.26 ± 9.21 (12.60)	15.20 ± 7.02 (16.37)	93.50	0.389	16.24 ± 7.91 (17.08)	13.64 ± 7.70 (12.60)	95.00	0.434	14.90 ± 7.65 (16.00)	14.21 ± 8.05 (14.15)	120.50	0.794
Procalcitonin	5.29 ± 5.36 (3.32)	7.26 ± 15.17 (1.38)	124.00	0.363	15.34 ± 20.86 (7.60)	3.31 ± 6.20 (0.89)	50.00	0.000*	10.14 ± 18.44 (4.06)	4.78 ± 8.78 (0.78)	94.50	0.017*
Uric acid	6.99 ± 2.86 (6.00)	6.46 ± 2.68 (6.10)	-0.70^	0.489	8.88 ± 2.07 (9.30)	5.77 ± 2.44 (5.30)	116.00	0.000*	5.80 ± 2.59 (7.00)	6.40 ± 2.80 (5.80)	412.50	0.349
Albumin	2.52 ± 0.40 (2.43)	3.08 ± 0.70 (3.02)	4.01^	0.000*	2.60 ± 0.43 (2.52)	3.06 ± 0.72 (3.01)	3.11^	0.003*	2.55 ± 0.47 (2.42)	3.16 ± 0.69 (3.10)	215.00	0.000*

**Figure 3:**
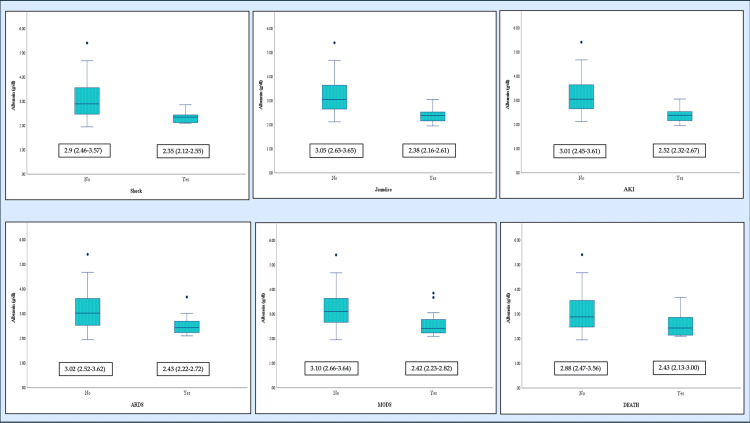
Significant association of serum albumin in complications such as shock (p < 0.005), jaundice (p < 0.005), AKI (p = 0.003), ARDS (p < 0.005), and MODS (p < 0.005), but not with mortality (p = 0.063) of scrub typhus patients. Median (IQR) is mentioned with each box and whisker plot. Results are expressed as median (IQR). P-value was calculated using the Mann-Whitney U test. ARDS = acute respiratory distress syndrome; AKI = acute kidney injury; MODS: multi-organ dysfunction syndrome

Multivariate analysis revealed important predictors. Patients with NLR between 7 and 17 had a 9.7-fold higher risk of ARDS (Table 2). They also had nearly 90-fold higher odds of MODS compared to those with NLR <3 (Table 2). Hypoalbuminemia was associated with all complications except encephalopathy (Figure 3, Tables 3-5), but independently predicted only MODS (Table 2). Hyperuricemia significantly increased the risk of encephalopathy (OR = 6.63, 95% CI = 1.25-35.12) and AKI (OR = 13.61, 95% CI = 3.33-55.68). Erythrocyte sedimentation rate (ESR) and C-reactive protein (CRP) were elevated in 48.5% and 100% of patients, respectively (Table 1). However, they were not associated with complications. In contrast, elevated procalcitonin was significantly associated with AKI and MODS (Tables 4, 5).

Table 3 summarizes the baseline characteristics of the study cohort. Female sex was significantly associated with mortality (p = 0.038). Several other parameters demonstrated statistically significant associations with death. These included total leukocyte count, neutrophil and lymphocyte counts, NLR, procalcitonin, serum urea, and uric acid. In contrast, CRP, bilirubin, transaminases, and creatinine, although frequently elevated, showed no significant association with mortality, nor did low platelet count. Neither the duration of fever at presentation nor the length of hospital stay correlated with outcome. Univariate analysis revealed significant associations between mortality and multiple complications (Table 2). Shock exhibited the strongest association (OR = 206.1, 95% CI = 18.61-29029.1), followed by ARDS (OR = 40.89, 95% CI = 4.53-368.35), encephalopathy (OR = 36.67, 95% CI = 5.67-211.77), MODS (OR = 19.5, 95% CI = 2.25-168.88) and AKI (OR = 4.48, 95% CI = 1.04-19.33). However, in multivariate logistic regression, only shock and encephalopathy emerged as independent predictors, increasing the odds of death by approximately 74-fold and 64-fold, respectively.

## Discussion

Our study determined the magnitude and distribution of serologically confirmed and clinically adjudicated scrub typhus cases from Raebareli and the adjoining districts. It also described their clinical profiles. While only sporadic cases had been reported from this region in the past decade, a recent increase in case burden was noted. Among 410 patients tested for scrub typhus IgM ELISA presenting with AUFI, 15.6% (64 patients) were positive. This seroprevalence was lower than reported in meta-analyses by Sondhiya et al. (26.41%) [5] and Tripathi et al. (20% in Uttar Pradesh) [15]. The lower seroprevalence in our study may reflect limited clinical suspicion among physicians. This occurs given the recent emergence and relative non-endemicity in this region, potentially resulting in under-diagnosed self-limiting scrub typhus cases. It may explain the sudden rise in the number of diagnosed cases of scrub typhus from 16 to 48 in the second year of our study due to the growing scrub typhus incidence in this non-endemic area, thereby increasing the clinical suspicion and higher pre-test probability. This finding carries significant public health implications, as scrub typhus is expanding in its geographical distribution. It is currently reported from 24 states and 86 districts in India, with the likelihood of further regions being added to this list [16]. Transmission potential is considerably higher in hot and humid conditions, with a marked predilection for the monsoon and post-monsoon seasons [17]. This seasonal trend was evident in our study, with most cases diagnosed between late August and early November. This duration matches the local proliferation of secondary scrub vegetation. Such vegetation provides suitable habitat for vectors, thereby increasing human exposure to trombiculid mite larvae [8,18].

Scrub typhus has conventionally been associated with agricultural activities in rural areas due to mite habitats [19]. However, our study demonstrated that a substantial proportion of cases (45.3%) originated from urban settings. This trend may reflect several factors. To name some, rampant construction, peri-urban gardening and farming practices, and climate change collectively provided a conducive environment for carrier rodents [20]. Additionally, under-reporting in rural populations cannot be excluded, given the lack of disease awareness and limited availability of diagnostic facilities outside urban centers. These findings highlight changing epidemiological trends and warrant further investigation into vector biology, dispersion of scrub vegetation, and tailored strategies to reduce transmission.

In our cohort, nearly three-fifths of patients were younger than 40 years. Male predominance was observed, likely reflecting the demographic profile of young farmers and students at higher exposure risk, while previous studies have not demonstrated a gender predilection [21,22]. Housewives were also prone to this disease due to the habit of open-field defecation, feeding the livestock, and maintaining the kitchen gardens. We observed age-related differences in complications. Younger patients (median age = 29 years) were more likely to develop ARDS, which may reflect strain-specific virulence. Older patients (median age = 51 years) exhibited increased AKI risk, potentially attributed to a decline in renal function with aging. Female patients demonstrated a higher risk of developing shock and ARDS in univariate analysis. However, this association did not persist after multivariate adjustment. Travel history to endemic regions was not contributory in any case. Pathognomonic eschars were identified in only three patients, localized to the groin and abdomen. They were not associated with any specific complications. This prevalence was considerably lower than reported in prior studies, where eschars were documented in 9.5% of patients in North India [23] and in 46-55% of cases in South India [11,24]. The regional variation in eschar prevalence may reflect strain diversity of *O. tsutsugamushi* and warrants further investigation.

Clinical manifestations in our cohort remained largely non-specific and overlapped with other AUFI. Laboratory evaluation, however, revealed characteristic trends which may aid in raising suspicion for rickettsial diseases when immediate confirmatory testing is unavailable [25]. Key laboratory findings included elevated transaminases. These typically did not exceed five times the upper limit of normal with an AST/ALT ratio >1. Thrombocytopenia and raised CRP were also common. Leukocyte counts ranged from normal to elevated. Notably, an NLR between 7 and 17 was independently associated with higher risks of ARDS, MODS, and mortality. This underscores its potential utility as a poor prognostic marker, especially as traditional inflammatory indices, such as CRP and ESR, showed no significant correlation with outcomes. Hypoalbuminemia also demonstrated strong associations with multiple complications and MODS. It can be attributed to systemic inflammation, increased protein catabolism, and impaired hepatic synthesis [26]. Albuminuria was observed in a substantial proportion of patients without alternative explanations. This may reflect vasculitic endothelial injury with enhanced capillary permeability. Alternatively, it may result from direct tubular damage impairing protein reabsorption [27]. However, it did not confer increased risk of AKI in our cohort. Hyponatremia and hyperuricemia were present in nearly half of the patients. These abnormalities occurred occasionally, even independent of AKI, which may reflect stress-related hormonal responses and activation of endothelial xanthine oxidase pathways that occur secondary to sepsis and hypoxia [28].

Despite confirmed scrub typhus, seropositivity for *Leptospira*, malaria, and dengue was observed in 14% of patients. An additional 11% showed equivocal results. Similar findings have been reported in previous studies, often attributed to either true co-infections or serological cross-reactivity, as clinical differentiation from scrub typhus remains challenging [29,30]. Confirmation with more specific modalities, such as polymerase chain reaction (PCR), would be ideal, though not readily available in our setting.

Apart from prodromal symptoms, the most frequent clinical presentation was community-acquired pneumonia (35.6%), typically with a non-productive cough. Due to limited awareness of scrub typhus, such patients were often initiated on empirical antibiotic regimens ineffective against the causative pathogen. This delay in treatment initiation leads to complications and referral to our tertiary care center. Notably, 17 of the 23 referred patients progressed to ARDS, with a case fatality rate of 47% despite adequate mechanical ventilation. This increased the odds of death by 41-fold, causing considerably higher mortality in our cohort than reported in prior studies, where ARDS was observed in 10-25% of cases with mortality rates ranging between 18% and 25% [11,12]. However, the lack of independent association between ARDS and mortality on multivariate regression underscores MODS as the terminal event driving adverse outcomes in all such patients.

The incidence of ARDS (26.5% vs. 18-23%) [11] and MODS (37.5% vs. 14-20%) [29,30] in our study exceeded previous reports, whereas the frequencies of shock (9.3% vs. 10.9%) and encephalopathy (14% vs. 9.5-25%) were comparable to earlier studies [11,21,23]. The overall mortality rate (14% vs. 7.69%) also exceeded the pooled mortality reported in a systematic review of Indian studies by Sondhiya et al. [5]. The increased incidence of MODS and higher case fatality observed in our study may reflect the presence of more virulent strains in this region, underscoring the need for genetic characterization of local isolates and evaluation of their antimicrobial resistance patterns to inform treatment guidelines. Complications such as hypotension requiring vasopressor support and encephalopathy warrant particular attention, given their independent association with mortality. Interestingly, neither fever duration at presentation nor length of hospital stay was associated with complications or mortality. This suggests potential variability in host immune responses and pathogen virulence and may have a genetic basis.

Our study’s strength lies in being the first to characterize the clinical and epidemiological profile of scrub typhus in this region. It highlights divergence from patterns reported elsewhere in India. These findings emphasize the importance of heightened clinical suspicion among physicians and the need for further research into strain virulence and antibiotic susceptibility. However, certain limitations must be acknowledged. The retrospective, hospital-based design largely comprised referred cases. This may not accurately represent community burden, as patients with milder or self-limiting illnesses may not have sought care. Additionally, some clinical records were incomplete. Diagnostic confirmation relied only on IgM ELISA rather than the gold standard IgM immunofluorescence assay or PCR, which were unavailable. Serotyping could not be performed, and diagnosis based on a fourfold rise in IgG titers was not feasible due to patients being lost to follow-up or financial constraints

## Conclusions

Our study highlights a concerning surge of scrub typhus in previously non-endemic regions of northern India, establishing it as an important cause of AUFI with frequent multi-organ involvement. Seasonal clustering during post-monsoon months was evident, with a peak in September and October. The study revealed a significant mortality risk, with an overall case fatality rate of 14%, exceeding rates reported from traditional endemic areas. Shock and encephalopathy emerged as independent predictors of mortality, while the pathognomonic eschar was present in only 4.7% of cases, complicating clinical diagnosis. Limited clinical awareness in non-endemic regions likely contributed to delayed recognition and treatment, which explains the higher complication rates and mortality observed in our cohort, exposing the lack of awareness. NLR, hypoalbuminemia, and hyperuricemia were discovered as laboratory predictors of complications. However, the true community burden remains uncertain due to under-reporting and under-diagnosis. The increased proportion of urban cases warrants surveillance beyond the traditional zone. Strengthening surveillance systems and integrating rickettsial screening into routine diagnostic protocols for AUFI across the state may improve early case detection. Conversely, in resource-limited settings, a pragmatic suspect-and-treat approach with doxycycline or azithromycin could help mitigate serious morbidity and mortality. This is particularly important given the non-specific clinical presentation and low eschar prevalence in this region. Further research should focus on genetic characterization of local strains, antimicrobial resistance patterns, and vector biology in emerging areas. Such studies would inform targeted prevention strategies and optimize treatment protocols for this re-emerging infectious disease.
